# Engineering bacterial motility towards hydrogen-peroxide

**DOI:** 10.1371/journal.pone.0196999

**Published:** 2018-05-11

**Authors:** Chelsea Virgile, Pricila Hauk, Hsuan-Chen Wu, Wu Shang, Chen-Yu Tsao, Gregory F. Payne, William E. Bentley

**Affiliations:** 1 Institute for Bioscience and Biotechnology Research, University of Maryland, College Park, Maryland, United States of America; 2 Fischell Department of Bioengineering, University of Maryland, College Park, Maryland, United States of America; 3 Department of Biochemical Science and Technology, National Taiwan University, Taipei, Taiwan; University of Illinois at Urbana-Champaign, UNITED STATES

## Abstract

Synthetic biologists construct innovative genetic/biological systems to treat environmental, energy, and health problems. Many systems employ rewired cells for non-native product synthesis, while a few have employed the rewired cells as ‘smart’ devices with programmable function. Building on the latter, we developed a genetic construct to control and direct bacterial motility towards hydrogen peroxide, one of the body’s immune response signaling molecules. A motivation for this work is the creation of cells that can target and autonomously treat disease, the latter signaled by hydrogen peroxide release. Bacteria naturally move towards a variety of molecular cues (e.g., nutrients) in the process of chemotaxis. In this work, we engineered bacteria to recognize and move towards hydrogen peroxide, a non-native chemoattractant and potential toxin. Our system exploits *oxyRS*, the native oxidative stress regulon of *E*. *coli*. We first demonstrated H_2_O_2_-mediated upregulation motility regulator, CheZ. Using transwell assays, we showed a two-fold increase in net motility towards H_2_O_2_. Then, using a 2D cell tracking system, we quantified bacterial motility descriptors including velocity, % running (of tumble/run motions), and a dynamic net directionality towards the molecular cue. In CheZ mutants, we found that increased H_2_O_2_ concentration (0–200 μM) and induction time resulted in increased running speeds, ultimately reaching the native *E*. *coli* wild-type speed of ~22 μm/s with a ~45–65% ratio of running to tumbling. Finally, using a microfluidic device with stable H_2_O_2_ gradients, we characterized responses and the potential for “programmed” directionality towards H_2_O_2_ in quiescent fluids. Overall, the synthetic biology framework and tracking analysis in this work will provide a framework for investigating controlled motility of *E*. *coli* and other ‘smart’ probiotics for signal-directed treatment.

## Introduction

**Fig 1:**
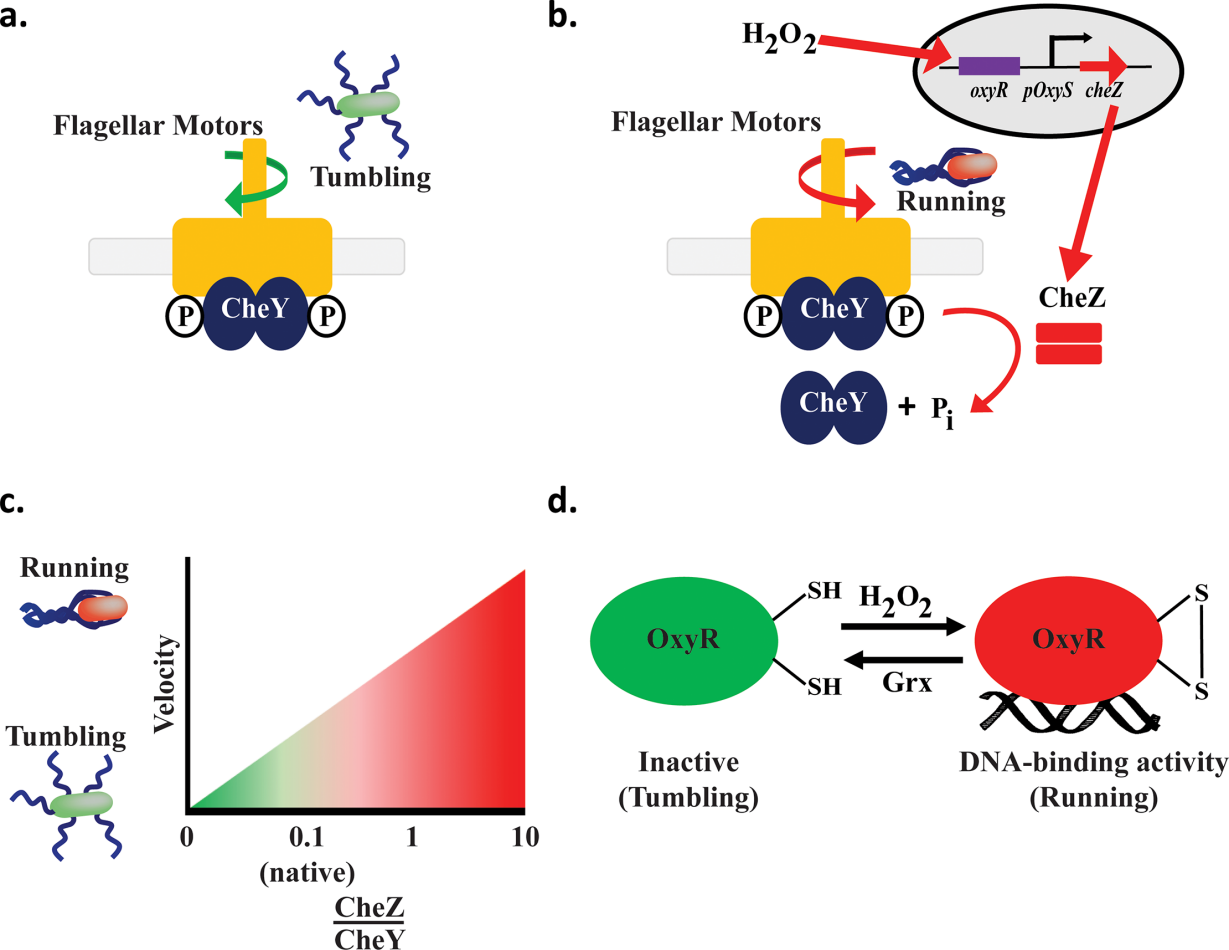
Hydrogen peroxide-controlled bacterial motility. *a*. *CheY activation*. Phosphorylated CheY binds to the flagellar motor complex, clockwise rotation resulting in tumbling. *b*. *CheZ activation and bacterial design*. Hydrogen peroxide modulates OxyR, enabling *oxyS* RNA activation of CheZ. CheZ dephosphorylates CheY, which is released from the flagellar motor resulting in counterclockwise rotation and a run. *c*. *CheZ-CheY ratio controls run/tumble*. As the ratio of CheZ to CheY increases (x axis), the bacteria decreases tumbling time and increases running time. In native *E*. *coli*, the ratio of CheY:CheZ expression is approximately 8:1 (42). *d*. *OxyR*-*ROS Receptors*. The OxyR transcription factor regulates the hydrogen peroxide oxidative stress response in *E*. *coli*. Hydrogen peroxide alters OxyR to activate OxyR DNA binding enabling *oxyS* RNA transcription. OxyS RNA is a global regulator. OxyR is reduced by intracellularly by glutaredoxin-1 (Grx) [5].

Bacteria naturally respond to oxidative stressors such as hydrogen peroxide and other reactive oxygen species (ROS) that are released by eukaryotic cells upon insult such as pathogen infection or wound generation. They possess several mechanisms, including the OxyR/S-mediated response triggered by hydrogen peroxide [1–3], for protection against toxicity. Eukaryotic and prokaryotic organisms also produce ROS as a byproduct of normal aerobic metabolism; thus, they have naturally developed machinery and mechanisms to convert the metabolic ROS side products into non-toxic products thereby maintaining homeostasis [2, 4]. At physiological levels of H_2_O_2_ (~20 nM), OxyR acts as a repressor of *oxyS* RNA transcription in *Escherichia coli*. [3, 5, 6]. OxyS RNA, in turn, is a global oxidative stress regulator mediating the activation or repression of over 40 genes [4, 7]. In the presence of elevated H_2_O_2_ levels, changes in the oxidation state of OxyR’s sulfhydryl groups at Cys199 and Cys208 promote the formation of a disulfide bond [2], that, in turn, modulates OxyR’s structural conformation leading to subsequent transcriptional activation of many promoters involved in oxidative stress regulon [5, 8, 9]. These include *oxyS* [4] (**Fig 1**). This structural change, involving oxidation and reduction reactions, occurs extremely quickly, at a rate of 9.7 s^-1^ [9].

In the gastrointestinal (GI) tract, mammalian cells produce elevated levels of reactive oxygen species in response to various wounds and diseases, such as intestinal bowel disease (IBD), ulcerative colitis (UC), and Crohn’s disease (CD) [10, 11]. We created a synthetic biology controller employing the *oxyS* promoter and its OxyR regulator within the native GI bacterium *E*. *coli*, as a ‘smart’ device for the eventual autonomous treatment of GI diseases based on wound-specific elicitation of ROS in general, and specifically, hydrogen peroxide.

OxyR’s sensitivity to H_2_O_2_ should allow it to be used as both a monitor of *E*. *coli’s* response to H_2_O_2_ and as a controller of gene expression [4, 9, 12–14]. Importantly, initial screens suggest that the H_2_O_2_ concentrations investigated here are within the reported physiological ranges for eventual deployment at wound sites, including those in the GI tract [15]. Notably, *in vivo* studies conducted in zebrafish, a popular vertebrate model system, demonstrated a burst release of H_2_O_2_ (0.1–0.2 mM) to recruit immune cells that serve to prevent infection [15]. Additionally, *in vivo* mice studies reported H_2_O_2_ levels of dermal wounds persisted during the immediate inflammatory days (> 200 μM; 2 days post-wounding) as well as post-inflammatory days (~150 μM; 5 days post-wounding) [16]. Given these indications, we performed studies to evaluate dynamics of the *oxyRS* promoter system, bacterial cell growth upon H_2_O_2_ insult, and our desired H_2_O_2_-mediated swimming phenotype.

Naturally, there are many considerations that will need to be addressed prior to an H_2_O_2_ –programmed “smart” probiotic therapy (e.g., control of product synthesis, delivery, persistence of probiotic strains, horizontal gene transfer, etc.). Even the most basic issues, such as the maintenance of the “programmable” functions of engineered bacteria that are deployed in noisy environments, should be considered [17, 18]. Nonetheless, several recent studies have explored the potential for “smart” bacteria to treat disease [19–25]. Also, Saeidi *et al*. noted the benefits of directing the therapeutic bacteria towards the site of action. As such, we and others have showed how exogenous triggers such as applied voltage from a microelectronic device [26], pathogen-released signal molecules [25], or heterogeneously synthesized bacterial autoinducers [27] could be used to “program” cell motility.

In this work, we turn to the endogenous, wound-indicating signal molecule, H_2_O_2_, as a directional cue; we fully recognize the potential conflict in that induced directionality of therapeutic strains could, in turn, lead to their own damage. In order to attract our engineered bacteria toward to a localized injury marked by the presence of hydrogen peroxide, we developed a system that guides *E*. *coli* swimming towards H_2_O_2_ by controlling the (i) ratio of run to tumble, and (ii) cell velocity in the presence of an H_2_O_2_ gradient. Bacterial motility regulator, CheZ, is a phosphatase responsible for controlling the level of phosphorylated CheY, which, in turn, regulates the tumbling mode of bacterial swimming [5, 9] (**Fig 1**). For this, *che*Z was expressed under control of the *oxyRS* gene-promoter system induced by the presence of H_2_O_2_ in *cheZ*^-^ *oxyRS*+ mutants; we retained the native *oxyRS* genes to prevent additional oxidative stress to the bacteria and deleted genomic *cheZ* to examine how exogenous H_2_O_2_ affects motility.

The CheY and CheZ phosphorylation and dephosphorylation cascade (in conjunction with methylation and demethylation cascades) activates the bacterium’s tumbling and running motions [5, 9], respectively (**Fig 1**). As noted above, elevated H_2_O_2_ activates OxyR [5] which then induces OxyS RNA transcription. Hydrogen peroxide concentrations in the 10^2^–10^3^ μM range causes toxicity to *E*. *coli* and decreases *E*. *coli* survival [28]. In physiologically relevant mice studies, researchers showed that a range between 0.1 to 0.3 mM of H_2_O_2_ was generated at the site of the ROS burst [29–31]. Hence, given the potentially 10-fold difference between the physiologically relevant and toxic levels, we hypothesized that engineered bacteria might tolerate, rapidly consume, and swim in response to increased H_2_O_2_ without suffering significant oxidative stress. Thus, such engineered H_2_O_2_-controlled bacteria may allow for a wide range of application for future therapies or applications.

In order to characterize the system, we measured *che*Z mRNA levels and performed various phenotypic experiments. We measured induced swarming using motility agar plates. Motility experiments were also conducted with both transwell plates and a microfluidic device; results were analyzed using Matlab [32] and ImageJ to determine a range of motility parameters. We found that with either no or low concentrations of H_2_O_2_ (0–50 μM) or very short H_2_O_2_ exposure times (e.g. < 5–10 min.), our engineered *E*. *coli* had no ability to swim, only tumble; however, with higher concentrations of H_2_O_2_ and/or longer induction times (>10–15 min.), we found cells actively swimming with long runs and with less tumbling. Over time, when cells swim in the presence of a CheZ-inducing molecular stimulant and do not swim in the absence of the same stimulant, there will be a net propensity for the persistence of cells in the presence of the stimulant. This is a process of pseudotaxis [21, 33–35] as directionality can be “programmed” for a signal molecule not specifically recognized by chemotaxis receptors and regulators, but by the controlled generation of a concentration gradient. The present system guides *E*. *coli* motility based on H_2_O_2_ control. Engineered bacteria were found to rapidly (within 5 to 15 min) express CheZ and swim in a dose-dependent manner to H_2_O_2_. While demonstrated using quiescent fluids, these studies which show rapid bacterial responses at physiologically relevant hydrogen peroxide levels, suggest that engineered commensal strains may prove beneficial in GI therapies using “smart” probiotics.

## Materials and methods

### DNA manipulation and growth conditions

The *E*. *coli* K-12 W3110-*ΔcheZ* strain (HCW01) was constructed using a one-step inactivation method [26]. The genetic constructs developed in this study were assembled using standard molecular biology protocols [36]. Briefly, the *oxyR* and *oxyS* gene-promoter sequence from *E*. *coli* corresponding to NC_010473:4256210–4257127 (+ strand) and NC_010473:42560054256114 (- strand), respectively and the genomic *che*Z gene were amplified using the primers specified in **S1 Table** and subsequently digested using the appropriate site restriction enzymes. The sequences relative to *oxyR* and *oxyS* promoter or *che*Z were ligated using T4 Ligase (New England Technologies). Ligations were transformed in *E*. *coli* Top10 cells (Invitrogen) and plated on LB agar plates supplemented with ampicillin (50 μg/mL) and incubated at 37°C. Plasmid DNA (**S1A Fig**) from each selected clone was isolated for sequencing using Qiagen miniprep and analyzed via restriction digestion. The same protocol was followed to insert eGFP into pET200 under the T5 promoter (**S1B Fig**). *E*. *coli* K-12 wild type (W3110) and *che*Z knockout strains were transformed with pFZY1 and pFZY1-*oxyR*-*poxyS*-*cheZ* (pHW02) or pFZY1, respectively.

Bacteria were grown in LB media supplemented with ampicillin (50 μg/mL) and incubated at 37°C in a shaker at 250 rpm for all growth experiments. For all overnight inoculations, bacteria were grown from glycerol frozen stock; all morning re-inoculations were adjusted with sterile media to OD_600_ 0.05. Except for motility plates, transwell assays and microfluidic assays, H_2_O_2_ induced experiments were conducted at 24°C at 250 rpm. The strain-plasmid nomenclature and shortened names are listed in **S1 Table**.

### Motility plates

*E*. *coli* W3110-pFZY1 (WT-pFZY1), W3110-*ΔcheZ*-pFZY1 (HCW01-pFZY1), and W3110-*ΔcheZ*-pFZY1-oxyR-*p*oxyS-cheZ (HCW01-pHW02) were grown to OD_600_ ~1.5, diluted to OD_600_ 0.1, and one 2μL droplet of cells (2x10^5^ cells) was added onto each motility plate. In turn, H_2_O_2_ (30% ACS-grade, Fisher Scientific, Pittsburgh, PA) was added to warm motility agar (Bacto Tryptone broth, BD Biosciences, Franklin Lakes, NJ with 0.5% NaCl, Sigma-Aldrich, St. Louis, MO and 0.25% agar, Fisher Scientific, Pittsburgh, PA) and poured into Petri dishes (100 mm diameter, 15 mL per plate) to yield concentrations of H_2_O_2_ (0–1 mM). Plates were incubated at 30°C for 18 hours. Negative and positive controls were performed in the absence and presence of 100 μM H_2_O_2_, respectively, added to the plates. For data analysis, technical and biological triplet data were obtained.

### Bacterial growth

*E*. *coli* WT-pFZY1, HCW01-pFZY1 and HCW01-pHW02 were inoculated into 25 mL of LB in 125 mL flasks. Bacteria were shaken at 37°C and sampled every 30 minutes until OD_600_ ~0.5. After induction, bacteria were shaken at either 24°C or 37°C and sampled every 15 minutes for two hours. For data analysis, technical and biological triplicate data were obtained.

### Hydrogen peroxide consumption

*E*. *coli* WT-pFZY1, HCW01-pFZY1 and HCW01-pHW02 were inoculated into 10 mL of LB in 50 mL flasks, and shaken at 37°C until OD_600_ ~0.5. Cells were pelleted and resuspended in fresh LB at OD_600_ 0.1 and 0.4. The bacteria were induced with 0–200 μM H_2_O_2_ in clear 96 well plates (total volume– 200 μL) at 24°C, 250rpm for 5–15 minutes. All samples were assayed for H_2_O_2_ consumption with standards using the Quantitative Peroxide Assay Kit (Pierce, Thermo Fisher Scientific, Waltham, MA). For data analysis, technical and biological triplet data were obtained.

### *cheZ* qPCR

*E*. *coli* WT-pFZY1, HCW01-pFZY1 and HCW01-pHW02 were inoculated into 5 mL of LB in culture test tubes, and shaken at 37°C until OD_600_ ~0.5. Cells were induced with 0.125–1 mM H_2_O_2_ at 24°C, 250rpm for 5–15 minutes and pelleted by centrifugation. RNA extraction was performed using TRIzol (Fisher Scientific) and samples were treated with Dnase I (New England BioLabs) to eliminate possible DNA contamination.

Quantitative PCR conditions were carried out on an Applied Biosystems 7300 Real-Time PCR system using a 2-step cycling protocol. Primers were used at a final concentration of 400 nM, and 10 ng of RNA was used as template in each 20-μl reaction. Each reaction was performed in triplicate, with outlying data removed for select samples. 16s rRNA was used as the endogenous housekeeping gene. To calculate the levels of *cheZ* expression ΔC_T_ values were calculated by the following equation: ΔC_T_ = C_T Target_-C_T Reference_. The ΔΔC_T_ value was calculated as ΔΔC_T_ = ΔC_T,sample_- ΔC_T,Wt_ where each ΔC_T_ are represented by the difference between the Target and Reference (16srRNA) values, as above. Also, the relative quantification (RQ) is calculated as 2^-ΔΔCT^. Error bars represent the standard deviation of each RQ (2^-ΔΔCT+s^ and 2^-ΔΔCT-s^). The relative quantification was based on the relative expression of *cheZ* versus 16S rRNA. Wild type *E*. *coli* C_T_ values for *cheZ* were used as reference for all samples.

#### CheZ quantification

*E*. *coli* WT-pFZY1, HCW01-pFZY1 and HCW01-pHW02 were grown overnight, reinoculated into 25 mL of LB in 125 mL flasks, and shaken at 37°C until OD_600_ ~0.5. Cells were induced with 0–300 μM H_2_O_2_ at 24°C, 250rpm for 5, 10, 15, and 60 min and then centrifuged at 4°C, 12,000 rcf for 10 minutes. Bacterial pellets were resuspended with 200 μL BugBuster (BugBuster HT, EMD Millipore) and protease inhibitor (HALT Protease Inhibitor Cocktail (100x), Fisher Scientific). Cell suspensions were shaken at 24°C, 150 rpm for 40 minutes. Insoluble cell debris were removed by centrifugation at 4°C, 12,000 rcf for 20 minutes and soluble fractions were transferred to new tubes.

Total protein concentration (Pierce BCA Protein Assay, Fisher Scientific) was calculated using the microplate procedure with BugBuster-BSA standards. Pre-stained ladder (Benchmark Prestained Protein Ladder, Fisher Scientific), His_6_-CheZ protein, and 25 μL of boiled samples (~110 μg total protein concentration) were loaded into 12% SDS-PAGE gels (Bio-Rad). A Semi-Dry Transfer Apparatus (Bio-Rad) was used to transfer proteins to nitrocellulose membranes (Thermo Scientific Pierce). The membranes were blocked with 10% milk (Blotting Grade Blocker Non Fat Dry Milk, Bio-Rad) overnight at 4°C. Membrane was washed three times using TBS-T buffer and incubated for 1h 30 min with 1:10,000 anti-CheZ polyclonal antibody (produced by New England Peptide). Before incubation with anti-CheZ, it was adsorbed in 25% lysed *E*. *coli cheZ* knockout strain extract, 5% Bovine serum albumin (BSA), Sigma Aldrich, St. Louis, MO) to decrease nonspecific binding. Membrane was washed three times using TBS-T buffer and incubated with 1:15,000 of anti-rabbit alkaline phosphatase antibody (Sigma Aldrich) solution 5% BSA, TBS-T). The membranes were developed for 1 hour at 24°C in development buffer (with BCIP/NBT), and the reaction was stopped using deionized H_2_O.

#### Motility videos

*E*. *coli* WT-pFZY1, HCW01-pFZY1 and HCW01-pHW02 (with pET200-T5-eGFP for trajectory images only) were inoculated into 5 mL of LB in 25 mL flasks, and shaken at 37°C until OD_600_ ~0.5. Bacteria were split into 1 mL cultures in culture test tubes and induced with 0 μM H_2_O_2_ (control) and 12.5–100 μM H_2_O_2_ at 24°C, 250 rpm for 5–15 minutes. The bacteria were centrifuged at 1,000 rpm and 4°C, washed twice, and resuspended in DPBS for brightfield and fluorescence motility videos (CellSense). Videos were recorded for 100 frames for subsequent Tumble Score [32] Matlab (version R2015a) analysis. For data analysis, technical duplicate and biological triplicate data were obtained.

#### Transwell motility assays

*E*. *coli* WT-pFZY1, HCW01-pFZY1 and HCW01-pHW02 were grown overnight, reinoculated into 5 mL of LB in 25 mL flasks, and shaken at 37°C until OD_600_ ~0.5. Cells were washed twice and resuspended in DPBS buffer to OD_600_ ~0.15. In a 6 well plate (Corning), 2.5 mL of cell suspension was added. In the top transwell, 1.5 mL of DPBS ± 0–300 μM glucose or 0–300 μM H_2_O_2_. Transwells were incubated at 37°C for two hours for the DPBS buffer and 25–300 μM glucose transwells; based on the H_2_O_2_ diffusivity properties dependent on temperature [37] and a Transwell Comsol model [27], the H_2_O_2_ transwells were incubated at 37°C for 45 minutes to ensure that the H_2_O_2_ gradient across the transwell membrane persisted throughout the experimental time course. For data analysis, technical duplicate and biological triplicate data were obtained.

#### Static gradient device

*E*. *coli* WT-pFZY1, HCW01-pFZY1 and HCW01-pHW02 were inoculated into 5 mL of LB in 25 mL flasks, and shaken at 37°C until OD_600_ ~0.5. Cells were centrifuged at 1,000 rpm and 4°C, washed twice, and resuspended in DPBS buffer. The bottom channel of the motility device [38] was pretreated with Pluronic F-127 (Sigma-Aldrich, St. Louis, MO) for 1 hour to minimize nonspecific retention of cells to channel walls. Before cell introduction, DPBS buffer was pumped into both source and sink channels using 1 mL syringe and syringe pump at a rate of 120 μL hr^-1^. Bacteria were grown until OD_600_ ~0.6–0.8 and then introduced into the bottom channel at the cell inlet. This initially loads bacteria for subsequent filming and study. Both ends of the bottom channel were then wiped and taped to stop flow. They are then exposed to a gradient introduced by providing fluids in the upper source and sink channels, each having different concentrations of the gradient solute. For traditional chemotaxis experiments, a glucose solution (1 mM in DPBS) was then introduced at a rate of 50 μl hr^-1^ to replace DPBS in the source channel and to establish a maximum gradient concentration of 100 μM. The sink channel was maintained with 1 mM DPBS. This methodology rapidly generates a glucose gradient within the bottom channel. Note that there is no flow experienced by the cells in the lower channel (for details, see Shang et al., 2017). The time when glucose was added to the source channel was set to be t = 0. Bright-field images and videos were taken in the middle of the bottom channel at 0, 10, 20 and 30 minutes by a 20X Olympus objective. Then, for pseudotaxis experiments, H_2_O_2_ solutions (0.5 or 3 mM; diluted in DPBS) were introduced using the same method as the glucose solution, with final maximum H_2_O_2_ concentration gradients of 50 and 300 μM. Bright-field images were taken at 0, 5, 10, 12, 16, 18, and 20 minutes by a 20X Olympus objective.

#### Statistical analysis

For most experiments, one-way ANOVA using a multiple comparisons' Tukey-Kramer post-test were performed using Matlab (version R2015a). α values of 0.05, 0.01, and 0.001 were used to indicate statistical significance. Data are reported as mean values and standard deviation of the error, unless otherwise stated. ANCOVA linear regression analysis was performed using Prism.

## Results and discussion

### Characterization of hydrogen peroxide controlled motility

**Fig 2:**
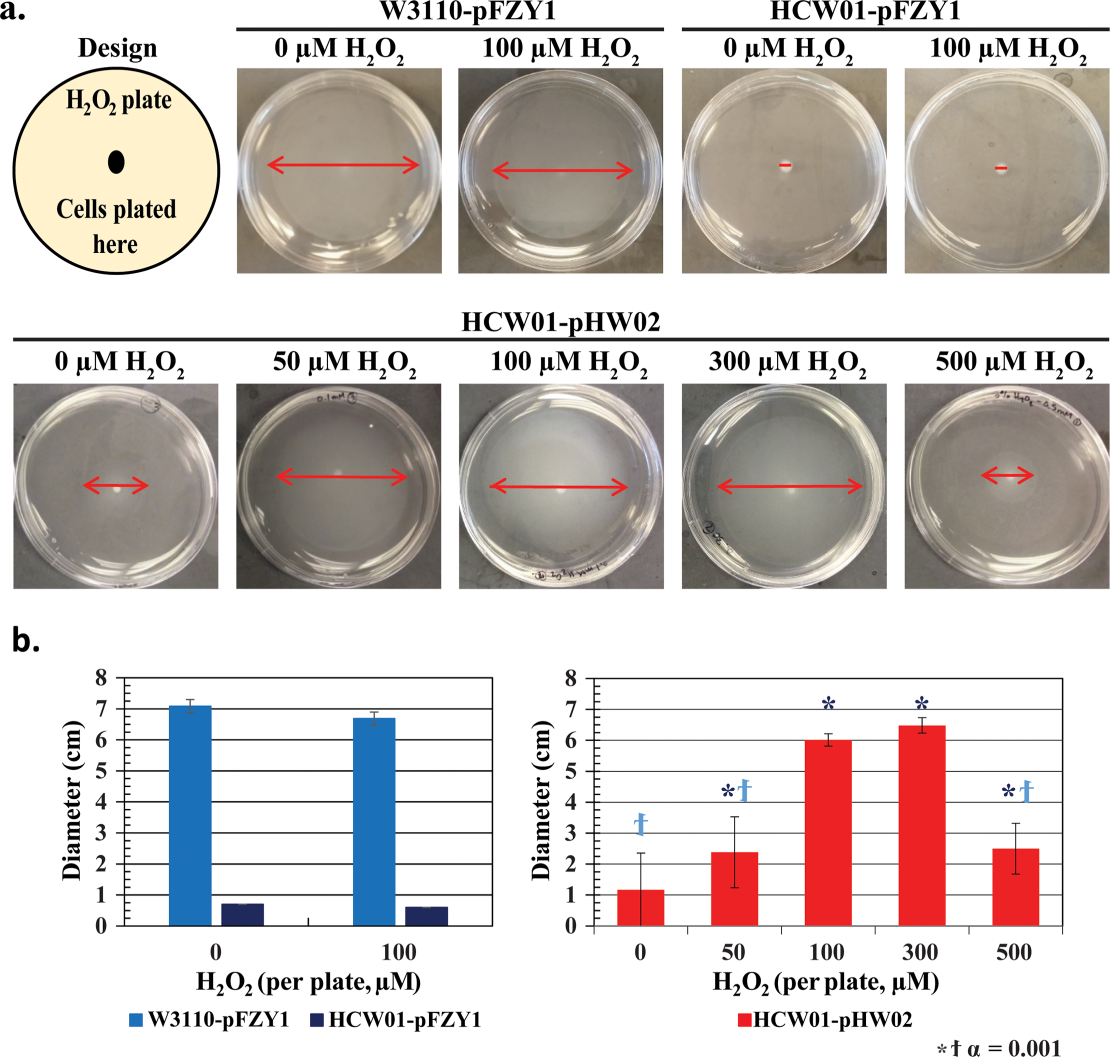
Bacterial swarming on motility agar exposed to varying concentrations of hydrogen peroxide. *a*. *Motility plates*. *Top Row*: WT-pFZY1 and HCW01-pFZY1 controls with 0 and 100 μM hydrogen peroxide-infused motility plates. Cultures were grown overnight. *Bottom Row*: HCW01-pHW02 with 0–500 μM hydrogen peroxide-infused motility plates. b. *Motility Spreading*. *(left) WT-pFZY1 and HCW01-pFZY1 exposed to 0 or 100 mM H*_*2*_*O*_*2*_. *(right) Quantification of HCW01-pHW02 with CheZ expression induced by H*_*2*_*O*_*2*_. Tukey-Kramer ANOVA and multiple comparisons analyses were performed with α = 0.001. ϯ indicates the samples differed significantly from WT-pFZY1. * indicates the samples differed significantly from HCW01-pFZY1.

Initial screening studies were carried out to test whether engineered cells exhibited enhanced motility in the presence of H_2_O_2_. In addition to evaluating whether a potential dose response could be obtained, we were interested to find at what concentration H_2_O_2_ would prove too toxic so as to permit enhanced motility. In **Fig 2A**, control experiments using wildtype W3110 cells with empty pFZY1 vector (WT-pFZY1) exposed to 0 or 100 μM H_2_O_2_ demonstrated a base case cell motility exhibited by ~7 cm rings with no obvious adverse reaction to the H_2_O_2_. Similarly, isogenic *cheZ* null mutants (HCW01) with empty pFZY1 vector exhibited no spreading in either case, with 0 or 100 μM H_2_O_2_. Then, using the same null mutants transformed with the *oxyRS* induced CheZ vector, pZY1-*oxyR*-*poxyS*-*cheZ*, (HCW01-pHW02) an increasing ring size was observed with increasing H_2_O_2_ from 0 to 300 μM (2.5–6.5 cm). Interestingly, we found that bacterial spreading decreased dramatically at 500 μM H_2_O_2_, presumably due to peroxide toxicity. Results from biological triplicates are indicated in **Fig 2B**. In addition, 1 mM H_2_O_2_ motility plates resulted in no center colony or spreading (data not shown). We note that the 0 μM plate, which had a minimal but non-zero spread, suggested that some CheZ activity enabled minimal motility, perhaps due to small levels of H_2_O_2_ through metabolic activity or simple read-through CheZ transcription and translation from our plasmid vector. Interestingly, the 0, 0.5 and 1 mM observations are consistent with previously published data [5]. Bacterial movement or “swarming” on motility plates does not depend on chemotaxis, rather it simply indicates search for nutrients [39–41]. In sum, our initial screening results are consistent with an hypothesis that H_2_O_2_-induced CheZ enables motility and that the induced motility rescues the wildtype swarming phenotype.

### Characterizing H_2_O_2_ uptake and effect on bacterial growth

**Fig 3:**
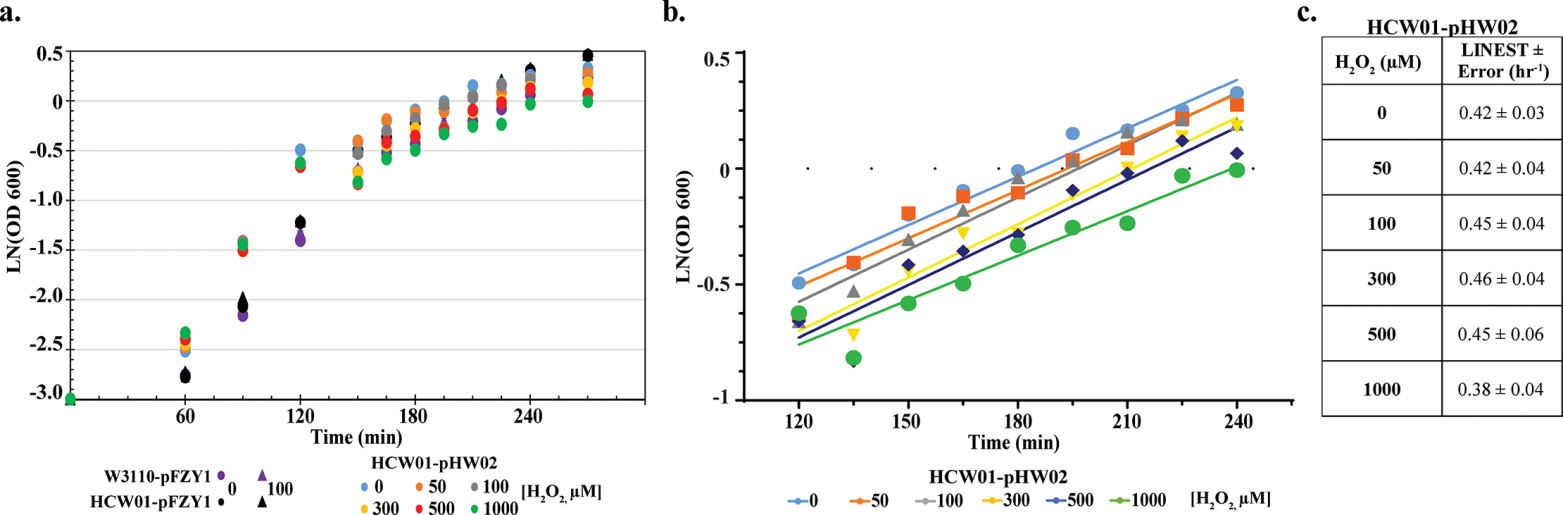
Effects of hydrogen peroxide on cell growth. *a*. *Cell growth curves*. WT-pFZY1 and HCW01-pFZY1 and HCW01-pHW02 vector at different levels of H_2_O_2_. *b*. *Cell growth curves following H*_*2*_*O*_*2*_ *addition*. HCW01-pHW02 growth with 0–1000 μM hydrogen peroxide induction concentrations. Lines indicated are least squares regressed best fits. *c*. *Linear regression analyses*. All linear regression analyses for HCW01-pHW02 were compared to 0 μM hydrogen peroxide.

We conducted growth experiments using WT-pFZY1, HCW01-pFZY1, and HCW01-pHW02 (H_2_O_2_-mediated *oxyS* transcription of CheZ). In **Fig 3A and 3B**, we grew cells at 37°C until 120 min when we added H_2_O_2_. At induction, the cultures were split to grow at 24°C and 37°C to examine how the temperature and H_2_O_2_ concentrations affect bacterial growth and H_2_O_2_ consumption. Regression analysis indicated that all preinduction growth rates were similar (data not shown). Then, analogously, after a short transient phase, cultures continuing after OD_600_ ~0.45 all grew at similar rates irrespective H_2_O_2_ for each temperature level (**Fig 3B and 3C** (24°C)**, S2A and S2B Fig** (37°C)) until H_2_O_2_ concentrations above 500 μM. We note that swarming experiments indicated reduced motility at concentrations above 300 μM, perhaps suggesting that swarming was more sensitive than cell growth to H_2_O_2_ at elevated peroxide levels. We note however, that swarming and growth experiments are quite different, particularly in that exposure to H_2_O_2_ occurs under different time scales.

**Fig 4:**
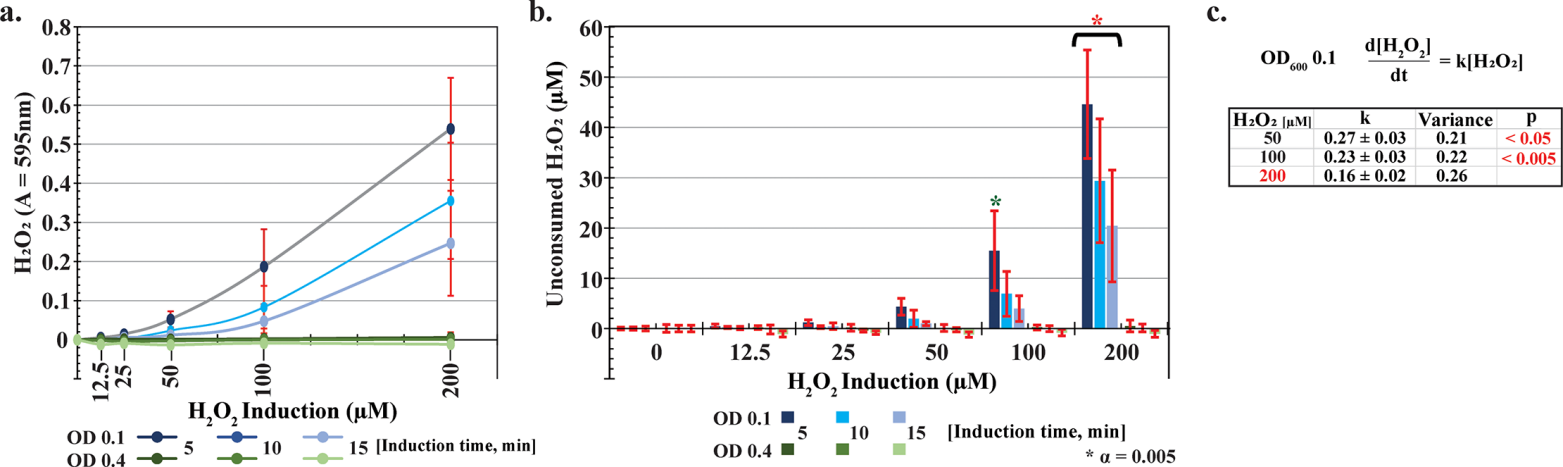
Hydrogen peroxide exposure and consumption. *a*. *Hydrogen peroxide levels*. WT-pFZY1 and HCW01-pFZY1 (controls) and HCW01-pHW02 induced with 0–200 μM hydrogen peroxide for 5–15 minutes at OD 0.1 and 0.4 were tested for supernatant H_2_O_2_ levels. H_2_O_2_ standard curves were developed (data not shown). *b*. *Calculated unconsumed hydrogen peroxide*. The unconsumed hydrogen peroxide for each sample was calculated. Tukey-Kramer ANOVA and multiple comparisons analyses were performed with * α = 0.005. * indicates the samples differed significantly from all samples. *c*. *Linear regression analysis*. Bacteria induced with 50–200 μM H_2_O_2_ (5–15 minutes) at OD_600_ 0.1 were analyzed using linear regression (Prism); first order decay rate constants were compared for statistical similarity.

We conducted H_2_O_2_ consumption experiments using the engineered bacteria (HCW01-pHW02) induced with 0 (control) to 200 μM H_2_O_2_. Specifically, cultures were induced at either OD_600_ 0.1 or 0.4 to gauge consumption levels at different cell concentrations after 5, 10, and 15 minutes of incubation with H_2_O_2_. At each timepoint, unconsumed H_2_O_2_ present in the cell media was measured. All cultures induced at OD 0.4 consumed the H_2_O_2_ very quickly, within 5 minutes (**Fig 4A and 4B**). When induced at OD 0.1, cells consumed high doses of H_2_O_2_ but more slowly. For example, in **Fig 4B**, roughly ~40 μM of 200 μM (1/5^th^) H_2_O_2_ had remained after 5 min, while ~5 μM remained at 5 min when dosed with 50 μM (1/10^th^). We approximated degradation kinetics using a first order rate law and estimated first order rate constants (see **Fig 4C).** As anticipated, the rate constant decreased with increasing hydrogen peroxide concentration. These results indicate that the higher concentrations H_2_O_2_ negatively impacted dissimilation ability of the affected cells.

Overall, these results indicate that the engineered bacteria rapidly consume H_2_O_2_. In addition to finding concordance with our initial motility studies, these results suggest that there is potentially flexibility in using H_2_O_2_ as a “smart” probiotic control signal. That is, the elicitation of H_2_O_2_ at a wound or other site could both activate engineered cells, but also be consumed by the same.

### H_2_O_2_-mediated *cheZ* transcription

**Fig 5:**
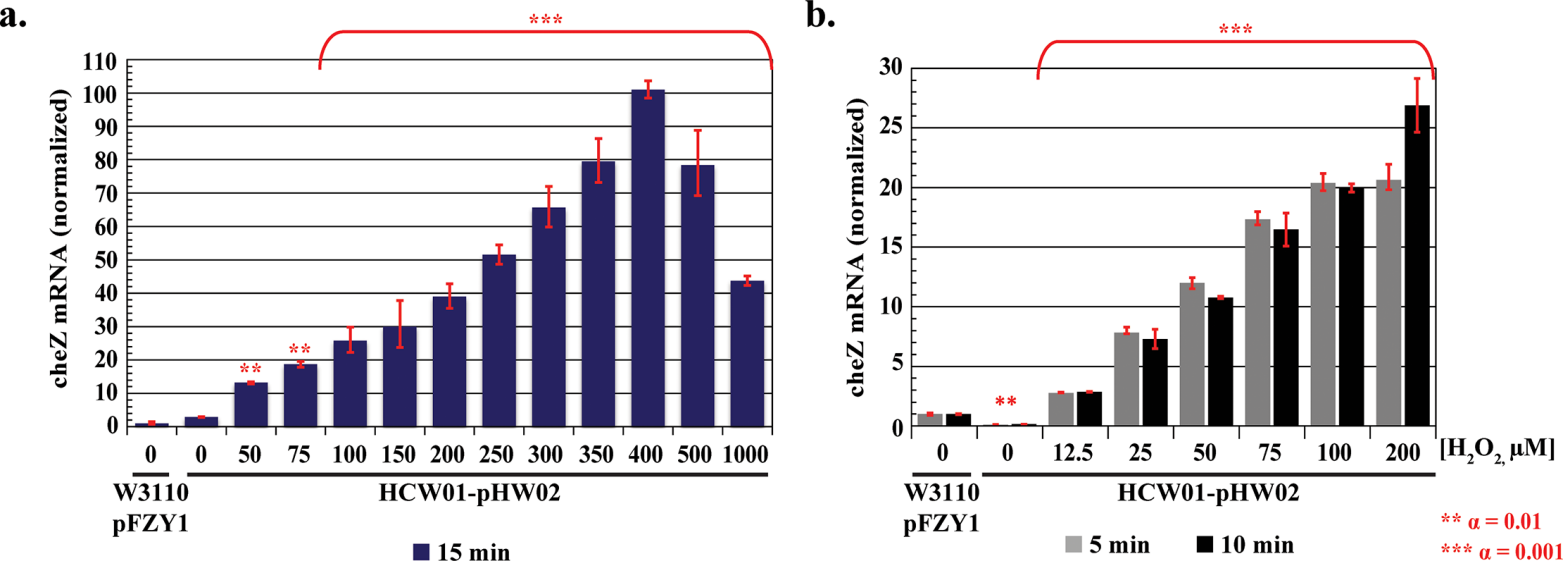
CheZ qPCR. *a*. *15 minute qPCR analysis*. *b*. *5–10 minutes qPCR analysis*. WT-pFZY1 is used as a control (i.e., genomic *cheZ* expression). All *cheZ* levels for HCW01-pHW02 were normalized to 0 μM hydrogen peroxide wildtype cells. Tukey-Kramer ANOVA and multiple comparisons analyses were performed with ** α = 0.01 and *** α = 0.001.

We conducted studies using qPCR to quantify *cheZ* expression. Data were normalized to 0 μM H_2_O_2_. For 15 minute H_2_O_2_ induction (meaning samples taken 15 min after introduction of H_2_O_2_ at various levels), qPCR data exhibited similar trends to our previous motility plate studies–a rise in gene expression from 0 to 300 μM and a decrease at 500 μM. In this case, to obtain greater resolution, we conducted additional tests at 350 and 400 μM, finding maximal *cheZ* mRNA levels at 400 μM 15 min post induction. Statistical analyses were performed by lumping tests above 100 μM and demonstrating significance relative to each of the lower levels (multiple comparisons one-way ANOVA, Tukey-Kramer post-test). Importantly, *cheZ* mRNA increased monotonically with H_2_O_2_ over time for all ranges tested (Fig 5A). A more detailed analysis using only 5 &10 min induction with 12.5–200 μM H_2_O_2_ showed similar increasing trends (Fig 5B). Interestingly, only 12.5 μM was needed to stimulate *cheZ* expression and this was observed at the first non-zero time point (5 min).

**Fig 6:**
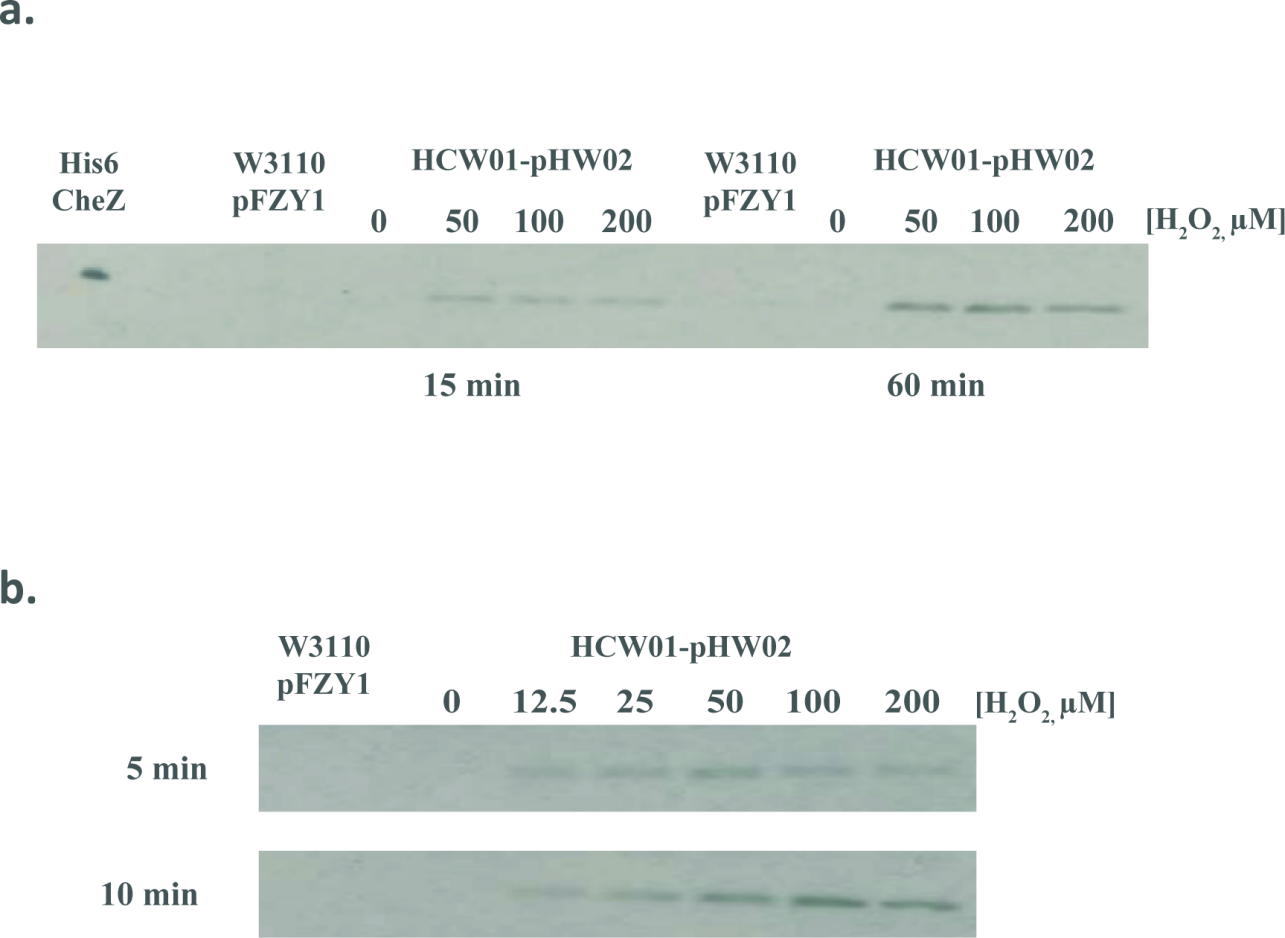
CheZ Western blot analyses. CheZ is expressed without a purification tag; Westerns are performed using rabbit anti-CheZ antibody (see Methods). *a*. *Hydrogen peroxide induction for 15 and 60 minutes*. *b*. *Hydrogen peroxide induction for 5 and 10 minutes*. His6-CheZ and WT-pFZY1 were controls.

In **Fig 6A and 6B**, we used Western blotting to correlate H_2_O_2_ with CheZ protein levels in the HCW01-pHW02 cells. At 15 min, CheZ levels increased with H_2_O_2_. We found peak levels at 50–100 μM; this tended to decrease at the higher H_2_O_2_ levels (although this was not a statistically validated trend) (**Fig 6A**). Interestingly, the CheZ from uninduced wildtype cells was not revealed, presumably below detection in these blots. Moreover, we found no CheZ bands in the uninduced HCW01-pHW02 cells. Subsequent studies were carried out at shorter induction times and at additional H_2_O_2_ concentrations (**Fig 6B**). Results were consistent and reveal that at 12.5 μM and 5 min H_2_O_2_ induction, there was significant CheZ present (significantly more than the wildtype cells). This level increased in time and in peroxide concentration until 100 μM above which perhaps H_2_O_2_ toxicity prevented further increases. These findings directly corroborated the relatively fast mRNA responses.

### H_2_O_2_-mediated motility

**Fig 7:**
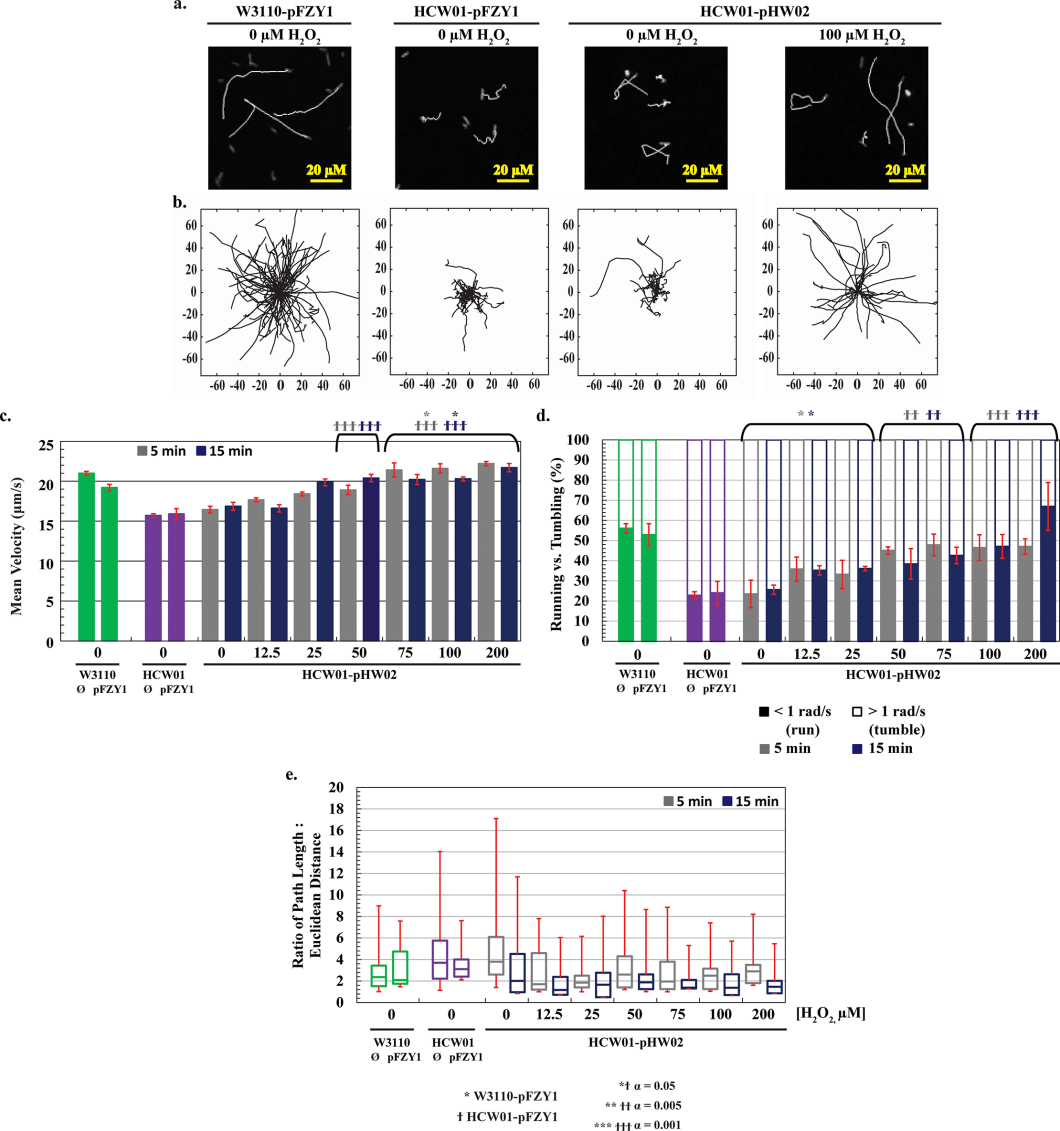
Characterization of hydrogen peroxide-induced motility. *a*. *Fluorescent trajectory images*. 5 second trajectories of fluorescent WT-pFZY1, HCW01-pFZY1, and HCW01-pHW02 (0 and 100 μM hydrogen peroxide) were mapped. *b*. *Rose graphs of trajectories*. 5 second trajectories are displayed from each trajectory’s origin to visualize the path lengths and angle changes. *c*. *Average velocities*. The average velocities of the control bacteria (WT-pFZY1, HCW01-pFZY1) versus the engineered bacteria (HCW01-pHW02; 0–200 μM hydrogen peroxide). *d*. *Percentage of time within trajectories of running vs*. *tumbling based on angle change*. Trajectories were calculated based on 5 second trajectories for all bacteria. ‘Running’ trajectories (solid bars) were based on the time it took the bacteria to move < 1 radian/s; ‘tumbling’ trajectories (lined bars) were based on the time it took the bacteria to move > 1 radian/s. *e*. *Ratio of path length*: *Euclidean distance*. The total distance traveled (path length) versus displacement (Euclidean distance) were calculated based on the initial and final points of 5 second trajectories for all bacteria. Data are presented as box and whisker plots with mean and quartiles indicated in box and extremums as whiskers. All data are quantified for 5 second trajectories. Tukey-Kramer ANOVA and multiple comparisons analyses were performed with * ϯ α = 0.05, ** ϯ ϯ α = 0.005, and *** ϯ ϯ ϯ α = 0.001. * indicates the samples differed significantly from WT-pFZY1. ϯ indicates the samples differed significantly from HCW01-pFZY1.

In order to analyze bacterial movements and to differentiate chemotaxis, pseudotaxis, and importantly, swimming from swarming (Fig 3), we carried out experiments in 2D using microscopy and cell motility videos. To simplify cell trajectory tracking, we transformed cells with a second plasmid constitutively expressing eGFP. In this way, non-motile cells could be differentiated from dust particles and debris. In Fig 7A, a few eGFP trajectories are depicted from representative traces. That is, a white trace line is produced as a cell moves from the beginning of its trajectory to its end. Similarities between the mutant and engineered cells without H_2_O_2_ were striking, as were the wildtype cells without H_2_O_2_ and engineered cells with 100 μM H_2_O_2_.

In **Fig 7B**, for cells without the GFP plasmid, Rose plots are depicted wherein many traces are superimposed with the initial points set at the origin of the coordinate axes. First, trajectories were observed in every direction as expected, illustrating random movement. Then, the *E*. *coli* HCW01-pFZY1 strain without H_2_O_2_ and *E*. *coli* WT-pFZY1 behaved as expected; *E*. *coli* WT-pFZY1 exhibited smooth running trajectories with random, interspersed tumbling while the *E*. *coli* HCW01-pFZY1 displayed increased tumbling with a few random spurts of running. Notably, the HCW01-pFZY1 cells moved far less from the origin than the WT-pFZY1 cells owing to the *cheZ* deletion and subsequent paucity of runs. Similarly, uninduced (0 μM H_2_O_2_) HCW01-pHW02 exhibited a phenotypic profile similar to HCW01-pFZY1. When induced with 100 μM H_2_O_2_, HCW01-pHW02 (**Fig 7A and 7B**) showed a phenotype more similar to WT-pFZY1, indicating that motility and CheZ running were recovered to wildtype levels.

To more directly compare the phenotypic responses, motility movies were again taken also without GFP-yielding plasmids (we had suspected increased burden due to the GFP expression). We used 5-sec trajectories and similar computational analyses, except that all stationary particles were discounted instead of just non-fluorescing particles. Quantified videos thus represent only moving cells. For statistical analysis, mean velocities were analyzed. We found HCW01-pHW02 induced with 50–200 μM H_2_O_2_ (5, 15 min. induction) had statistically higher (α = 0.001) velocities than the HCW01-pFZY1 control (the two control bars represent 5 & 15 min samples). Also, CheZ induction with 75–200 μM H_2_O_2_ for both 5 and 15 minutes showed significant (α = 0.05) increases compared to the WT-pFZY1 cells with velocities reaching ~23 μm/s and 19 μm/s, respectively. HCW01-pHW02 with 0 μM H_2_O_2_ were observed to have similar velocities to the HCW01-pFZY1 cells (~16.5 μm/s, **Fig 7C**). Velocities were observed to increase monotonically from 12.5 μM to 75 μM H_2_O_2_. In **S3B Fig**, we demonstrate that the same conclusions were drawn using both mean and median velocities.

Another quantity used to characterize swimming is the net angle change as a cell moves from frame to frame. For 5 second trajectories, the percentage of trajectories that exhibited a net angle change of < 1 radian/s and > 1 radian/s [42] were measured; this is calculated by resolving the direction and angle change between subsequent frames and averaging over the 5 second trajectory to classify the bacteria as “running” or “tumbling”, respectively (**S3A Fig**). Swimming percentages, which we denote the fraction of time running versus tumbling, were calculated to be ~20–25% for the HCW01-pFZY1 cells and the uninduced HCW01-pHW02. The percent swimming observed for the HCW01-pHW02 induced with 12.5 to 25 μM H_2_O_2_ were notably higher (~35%). At concentrations above 50 μM H_2_O_2_, cells were observed to have increased running (~40–48%, solid bars, **Fig 7D**). Conversely, the increase in percent running per trajectory was inversely proportional to the average angle change per trajectory (**S3A Fig**). The increased velocity observed with H_2_O_2_ is consistent with the increased fraction of running within a trajectory.

Interestingly, we observed that CheZ protein expression increased nearly monotonically with H_2_O_2_ concentrations until ~100 μM, which mirrored the calculated swimming velocity and percentage of run versus tumble. At H_2_O_2_ levels above ~100 μM, the CheZ level appeared to drop while the swimming parameters appeared to plateau. Also, the swimming parameter levels appeared to ultimately match the WT phenotype. This was contra-indicated by the *cheZ* mRNA levels which increased monotonically to ~ 400 μM, suggesting other cellular control mechanisms override continued increases in *cheZ* mRNA.

We did note, however, that the lower concentrations of H_2_O_2_ yielded higher *variability* in *cheZ* phenotypic expression within the population. For example, we found greater ranges in the standard deviations (25 and 75% quartiles) and extremums in the ratio of path length to Euclidean distance at the lower levels (**Fig 7E**). This measure is the ratio of the sum of all discrete distances travelled and the net distance from the beginning to end of a trajectory. It had significant variability and thus was an interesting measure to characterize the population distribution and its evolution with H_2_O_2_. In general, with increasing H_2_O_2_ concentration and induction time, the heterogeneity among phenotypes within the overall population decreased towards the observed wildtype values. Similar phenotypic focusing phenomena were observed in response to addition of quorum sensing signaling molecules [43].

### Chemotaxis and pseudotaxis

#### Transwell apparatus

**Fig 8:**
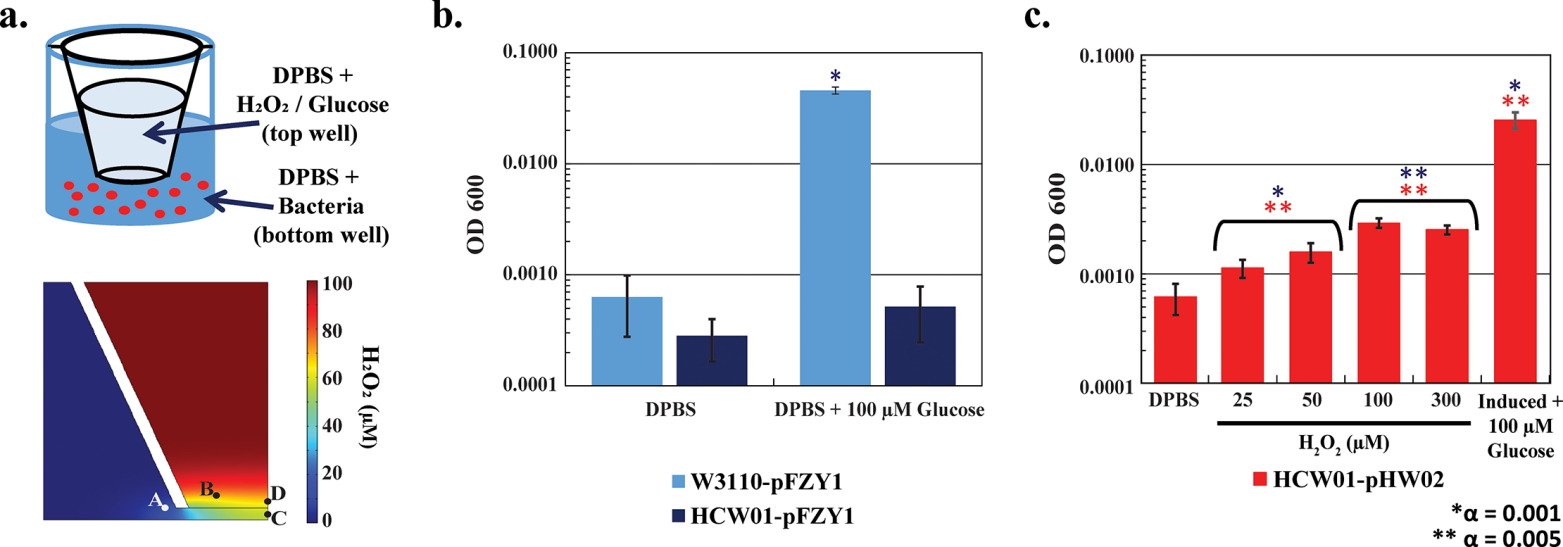
Chemotaxis versus pseudotaxis bacterial response. *a*. *Transwell scheme and simulated transwell gradient*. Bacteria were suspended in DPBS in the bottom of the transwell apparatus. DPBS, DPBS + 100 μM glucose or DPBS + 100 μM hydrogen peroxide solutions were loaded into the top of the transwell apparatus for testing. DPBS in both top and bottom served as random motility controls. Chemotaxis (towards glucose), or pseudotaxis (towards hydrogen peroxide), respectively, were also determined. *b-c*. *Quantification of bacterial motility*. For chemotaxis, the bacteria cell number was assayed in the upper chamber after 2 hours; for pseudotaxis, the bacteria cell number was assayed in the upper chamber after 45 minutes. Tukey-Kramer ANOVA and multiple comparisons analyses were performed with * α = 0.001 and ** α = 0.005. Blue * indicates the samples differed significantly from HCW01-pFZY1 + DPBS. ** HCW01-pHW02 + DPBS.

Transwell motility assays (Fig 8A) allow for direct observation of free swimming in 3D, and more specifically, initial analysis of chemotaxic versus pseudotaxic responses. We used Comsol to provide predicted H_2_O_2_ concentration gradients over time at 37°C for different initial H_2_O_2_ levels. At time zero, H_2_O_2_ was assumed to be uniform within the upper chamber, which in turn was assumed quiescent (no flow). In S4 Fig, we have indicated the predicted concentrations at points A-D over time. By examining concentrations at various locations relative to each other (e.g., difference between A&B, or C&D), our simulations suggest that the H_2_O_2_ gradients persisted over the course of the subsequent swimming experiments (S4B Fig).

We suspended exponentially growing bacteria in the bottom of a transwell apparatus containing Dulbecco's phosphate-buffered saline (DPBS) and at time zero, introduced either H_2_O_2_ or glucose in order to evaluate the swimming of bacteria vertically upward into the upper chamber. Glucose represents a chemotaxis positive control. By analyzing starting conditions with bacteria only present in the lower chamber and ending conditions with swimming bacteria in the upper chamber, we evaluated the extent to which bacteria swim to a chemoattractant such as glucose. These studies are analogous to our previous work [27] wherein bacteria were found to swim towards quorum sensing autoinducer AI-2, another known chemoattractant. In pseudotaxis, if the run of a tumble/run scheme is more frequent and/or of longer duration in the presence of a molecular stimulant, then on average the bacteria will accumulate in the direction of the stimulant [21, 34]. In our tests, statistical analysis (multiple comparisons one-way ANOVA, Tukey-Kramer post-test) shows significant differences from WT-pFZY1 with DPBS (control) in the upper chamber compared with WT-pFZY1 with glucose (chemoattractant) in the upper chamber (**Fig 8B**). Additionally, we found that HCW01-pHW02 induced with H_2_O_2_ migrated to the upper chamber in an apparent dose-dependent manner (**Fig 8C**). Importantly, while the pseudotaxic response to H_2_O_2_ was notably lower than the chemotaxic response to glucose, the result that cells swam to the upper chamber was statistically relevant (e.g., different than random swimming). This is particularly noteworthy in that previous findings have shown that bacteria swim *away* from H_2_O_2_ [44]; however, our findings suggest that rather than swim away from the potentially toxic ROS molecule, the bacteria instead swam towards H_2_O_2_. When considering our results from **Fig 3**, we suspect that these cells metabolize or dissimilate the potential toxin at the same times they were found to swim towards it. To our knowledge, this is the first such report, and this suggests that pseudotaxis can be controlled by internally regulating the CheZ level via externally applied H_2_O_2_.

#### Microfluidic device

**Fig 9:**
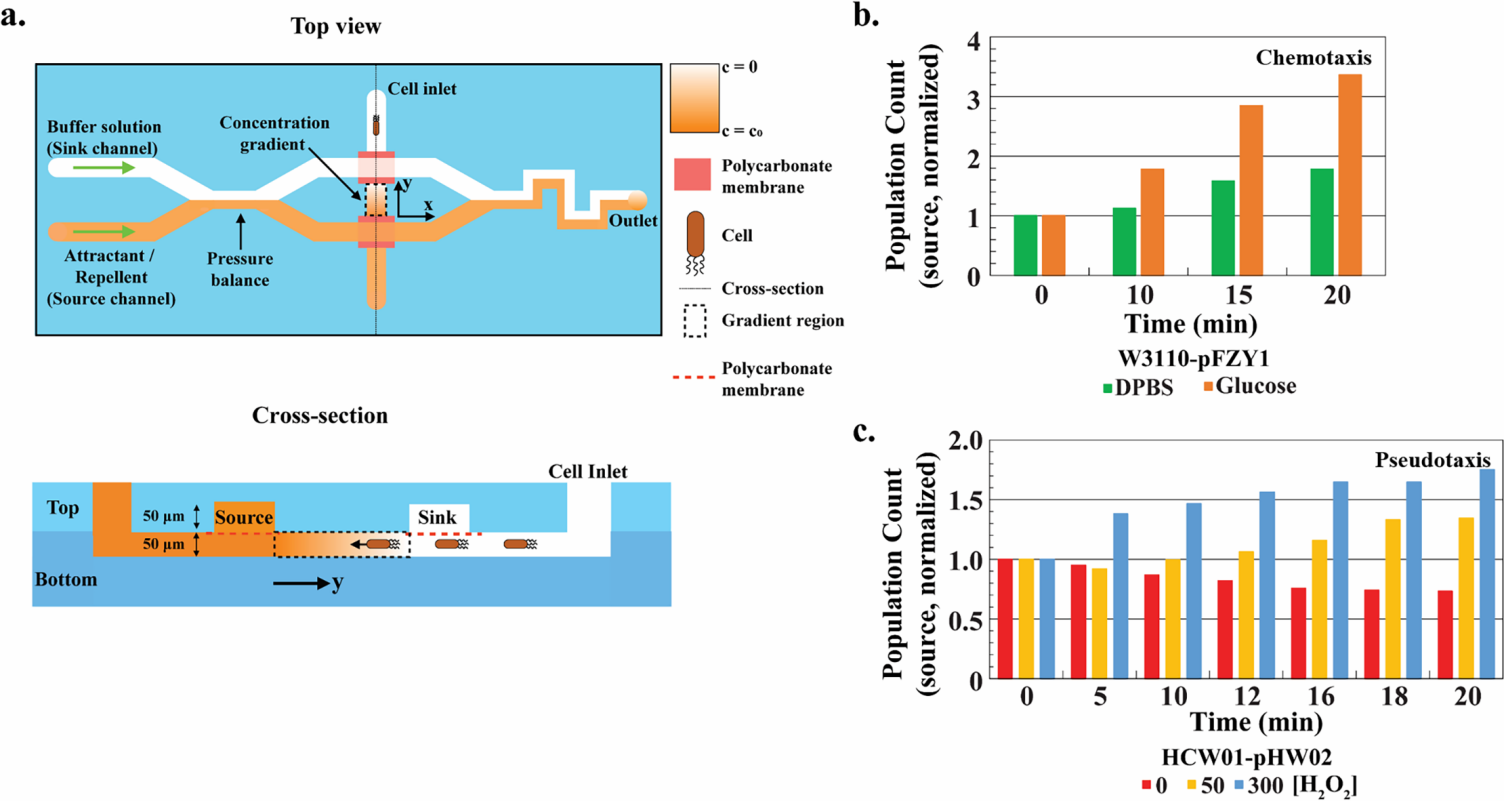
Pseudotaxis using a gradient-generating microfluidic device. *a*. *Microfluidic device scheme* [38]. Cells are introduced to the cell inlet (Top view) which places cells in the bottom channel (Cross-section). The chemical gradient is established by introducing buffer and attractant in flow at sink and source locations, respectively (see Methods). Cells are imaged near source, sink, and in the middle of the gradient (Top view). *b-c*. *Chemotaxis vs*. *pesudotaxis population source image analysis*. Images were taken at the indicated time intervals with t = 0 designated to be the introduction of glucose or H_2_O_2_ solution into the source channel. All population counts normalized to time 0 population counts at the respective positions. WT-pFZY1 with 100 μM glucose as the source yielded a chemotaxic response (towards glucose source). HCW01-pHW02 with hydrogen peroxide demonstrated pseudotaxis.

While transwells provided a quantitative method for determining globally the directional responses to glucose and H_2_O_2_, we performed additional studies using a microfluidic motility device [38] to discriminate more precisely the cellular responses due to chemical gradients. That is, using a well-controlled microfluidic device that employes a well-controlled and more uniform concentration gradient enables one to anaylze the bacteria’s phenotypic response both visually and analytically on a per cell basis so that cell and system design parameters for future applications can be estimated more accurately. As noted in Methods, bacteria were introduced into the bottom channel of a dual chamber device at the cell inlet (Fig 9A, top view). These cells are localized generally at the inlet but are free to disperse within the bottom channel (Fig 9A, cross-section). As soon as cells are added, the ends are taped to stop flow. Immediately thereafter, DPBS buffer is introduced into the sink channel of the top layer and either glucose or H_2_O_2_–supplemented DPBS solution was added to the source channel (Fig 9A) which started the experiment (t = 0). The concentration gradient of solute (glucose or H_2_O_2_) is established in the area indicated by the y-axis arrow (Fig 9A, top or cross section views). The cells in this lower channel are then free to swim according to the emerging gradient. A full description of the device and established gradients is available [38].

At appropriate time intervals, images near the sink, middle, and source were taken to represent the prevailing population and cell density. In **Fig 9B**, we quantified the WT-pFZY1 chemotaxic response to glucose. Based on data normalized to the cell numbers at time 0 and at the source, we observed a slight increase over time in the source population for WT-pFZY1 with DPBS (no attractant). As we presume there may have been more cells at the source end of the device (closest to the inlet), this represents the natural spreading of the population to unpopulated regions. Then, when the cells were exposed to a glucose gradient, we observed a far greater increase in population at the source. This increase was observed to be sustained for over 20 min. Analogously, in **Fig 9C**, we examined swimming when bacteria were exposed to H_2_O_2_. When pHW02 were exposed to increasing amounts of H_2_O_2_, we observed an increase in the number of bacteria found at the source (**Fig 9C**). Moreover, we found that in only 5 minutes exposure to a 300 mM H_2_O_2_ gradient, a notable increase in bacteria was found. Consistent with our previous results, the cells exposed to lower H_2_O_2_ concentrations (50 μM) responded more slowly and to a lesser extent. These results again demonstrate that CheZ-engineered bacterial cells can be “programmed” to swim towards H_2_O_2_. We presume that as cells moved towards the H_2_O_2_, they also helped to dissipate the level, enabling additional cells behind to swim towards the increasing peroxide level. We did not test, however, such spatially resolved H_2_O_2_ levels.

In the current construct, because cells do not swim (or swim less) in the absence of the hydrogen peroxide, there should be a greater propensity for the cells to accumulate in the desired locale. An advantage to this system is that there is minimal competition with native chemotaxis processes (e.g., directionality to glucose or other strong chemoattractants). That is, that upon actuation of CheZ expression, the CheZ mutant should have fully restored motility and will swim according to the prevailing cues until the cell leaves the region of elevated H_2_O_2_.

## Conclusion

We constructed a simple synthetic H_2_O_2_-responsive promoter system and used the system to guide bacterial swimming. Complementary studies [6, 17, 45, 46] demonstrate that additional regulatory constructs based on engineered plasmid controllers and/or engineered host cells enable tight or “tuned” control of gene expression based on signal molecule concentration. That is, the current system restores swimming motility while only minimally altering native circuitry.

We employed population based and single cell based analytical methods to characterize the system that enable *E*. *coli* to sense and pseudotax towards H_2_O_2_ in highly-controlled and defined manners. Studies of CheZ expression and motility revealed a practical threshold level of ~12.5 μM was suitable for rapid and sustained induction of CheZ and cell swimming. We noted also that the bacteria were able to quickly consume or otherwise dissipate H_2_O_2_, diminishing its effects so that over a putative therapeutic range, bacteria exhibited little to no growth inhibition.

In physiologically relevant settings (e.g., GI tract), reported H_2_O_2_ concentrations in mice studies were > 200 μM during the inflammatory phase and ~150 μM during the post-inflammatory phase in dermal wounds [47]; additional mice studies have shown that a range of 100–300 μM of H_2_O_2_ was generated at the site of the ROS burst release [29–31]. Correspondingly, our studies have indicated engineered cells functioned satisfactorily when exposed to H_2_O_2_ levels up to the 100–300 μM range, experiencing diminished CheZ expression, growth and motility at much higher levels (~500 μM). Thus, we suggest that the synthetic biology framework and tracking analysis in this work could be applied for the detailed design of engineered probiotics that would be deployed for directed treatment in the GI tract.

## Supporting information

S1 Fig Plasmid designs. a. Plasmid design of pFZY1-oxyR-poxyS-cheZ. b. Plasmid design of pET200-t5-eGFP.
(TIF)

S2 Fig Growth curves, incubation at 37°C. *a*. *Post-induction growth curves*. WT-pFZY1 and HCW01-pFZY1 with and without 100 μM hydrogen peroxide were controls. *b*. *Tabulated specific growth rates*. HCW01-pHW02 growth with 0–1000 μM hydrogen peroxide induction concentrations. All linear regression analyses for HCW01-pHW02 were compared to 0 μM hydrogen peroxide.
(TIF)

S3 Fig Phenotypic expression of hydrogen peroxide-induced motility. *a*. *Average angle change*. The average angle change in degrees per 5 second trajectory of the control bacteria (WT-pFZY1, HCW01-pFZY1) versus the engineered bacteria (HCW01-pHW02; 0–200 μM hydrogen peroxide). These values are inversely proportional to the percent running per trajectory. *b*. *Mean vs*. *median velocity*. Quantification and comparison of reported mean vs. median velocities for all bacteria. Slightly lower velocity and higher variability are associated with the median velocity. Median and mean velocities follow similar trends. ϯ (α = 0.05) indicates the samples differed significantly from HCW01-pFZY1.
(TIF)

S4 Fig Simulated hydrogen peroxide levels (Comsol). *a*. *Comsol model heat maps*. Four points (A-D) were recorded to estimate various hydrogen peroxide concentration gradients over time. Heat gradient maps were constructed for 45 minute (*left*) and 90 minute (*right*) gradients. Heat maps were generated using 50, 100, and 200 μM hydrogen peroxide starting concentrations. *b*. *Hydrogen peroxide gradient curves over time*. For each point (A-B, *left*; C-D, *right*), the hydrogen peroxide gradients were calculated over time to determine dynamics, steady-state conditions, and optimal measurement times (45 minutes).
(TIF)

S5 Fig Uncut CheZ Western blot images. *a*. *Hydrogen peroxide induction for 5 minutes*. *b*. *Hydrogen peroxide induction for 10 minutes*. *c*. *Hydrogen peroxide induction for 15 and 60 minutes*. His6-CheZ and WT-pFZY1 were controls.
(TIF)

S1 Table Strains designs and primer designs of pFZY1-oxyR-poxyS-cheZ. (TIF)
