# Supplementary figures and images for: Engineering bacterial motility towards hydrogen-peroxide
Source: PLoS One. 2018 May 11;13(5):e0196999. doi: 10.1371/journal.pone.0196999 (PMC5947916; doi:10.1371/journal.pone.0196999)

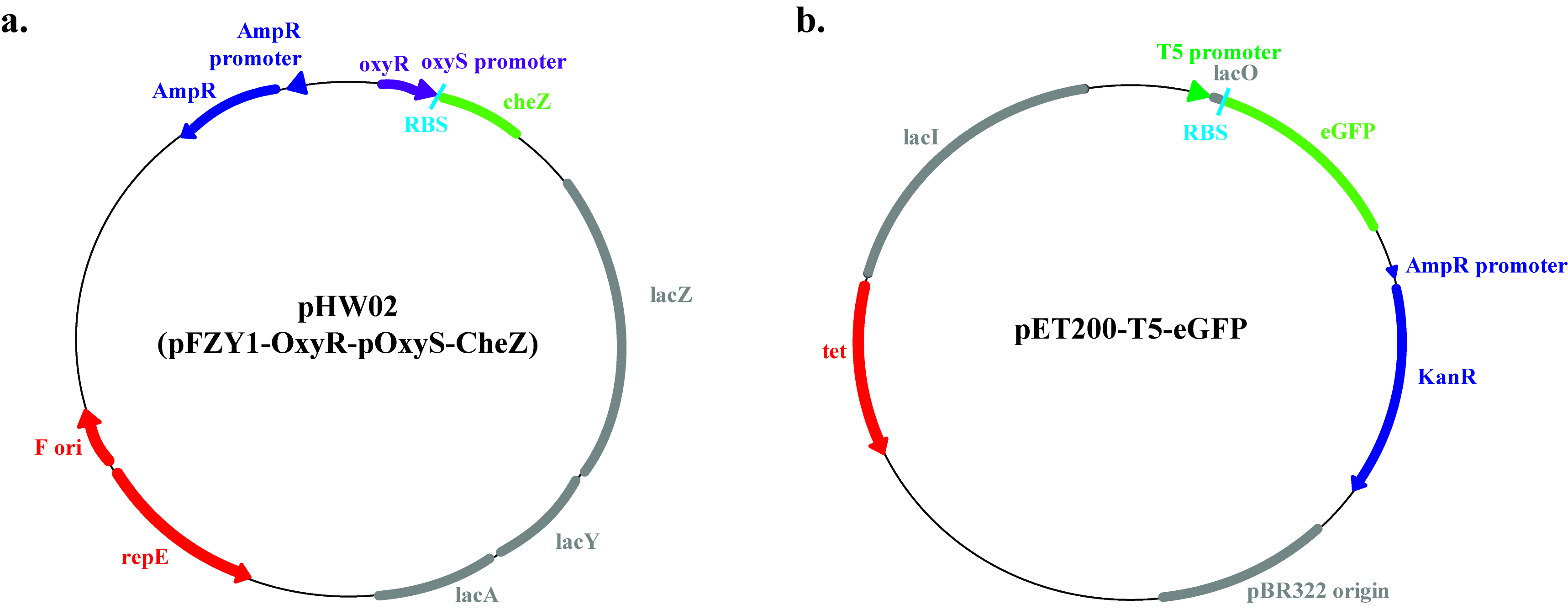

Supplement: S1 Fig — a. Plasmid design of pFZY1-oxyR-poxyS-cheZ. b. Plasmid design of pET200-t5-eGFP. (TIF) [file pone.0196999.s001.tif]

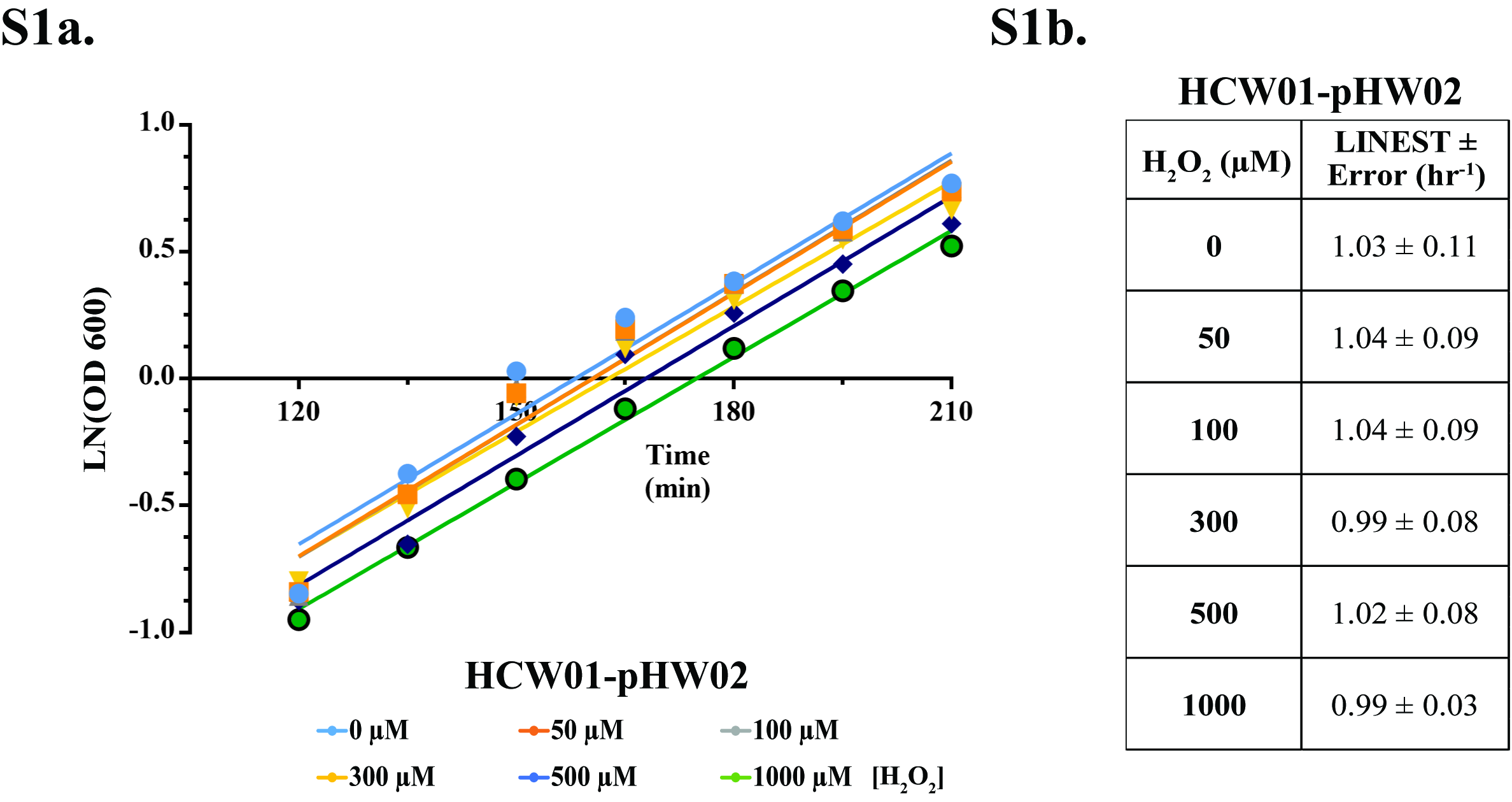

Supplement: S2 Fig — a. Post-induction growth curves. WT-pFZY1 and HCW01-pFZY1 with and without 100 μM hydrogen peroxide were controls. b. Tabulated specific growth rates. HCW01-pHW02 growth with 0–1000 μM hydrogen peroxide induction concentrations. All linear regression analyses for HCW01-pHW02 were compared to 0 μM hydrogen peroxide. (TIF) [file pone.0196999.s002.tif]

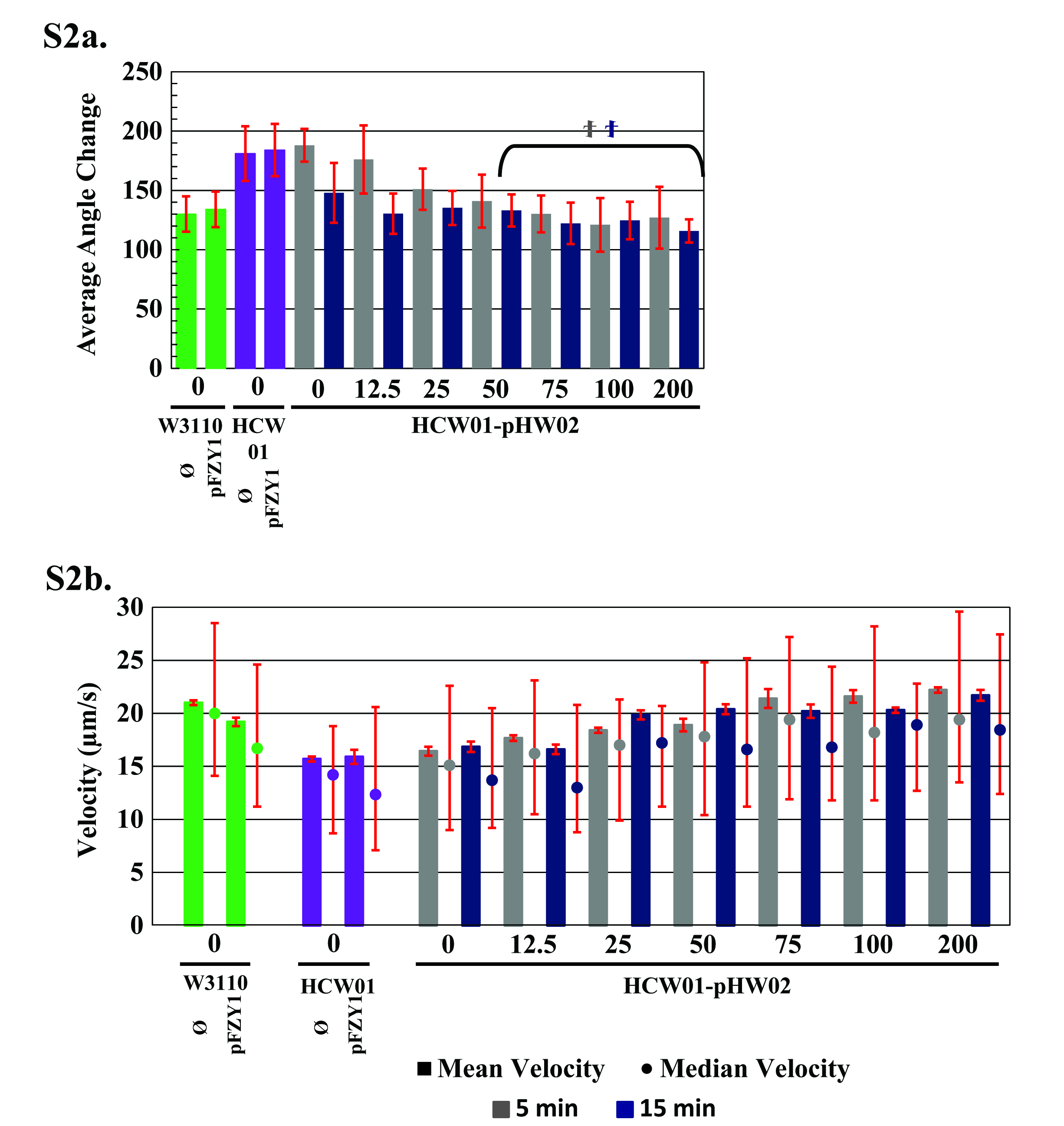

Supplement: S3 Fig — a. Average angle change. The average angle change in degrees per 5 second trajectory of the control bacteria (WT-pFZY1, HCW01-pFZY1) versus the engineered bacteria (HCW01-pHW02; 0–200 μM hydrogen peroxide). These values are inversely proportional to the percent running per trajectory. b. Mean vs. median velocity. Quantification and comparison of reported mean vs. median velocities for all bacteria. Slightly lower velocity and higher variability are associated with the median velocity. Median and mean velocities follow similar trends. ϯ (α = 0.05) indicates the samples differed significantly from HCW01-pFZY1. (TIF) [file pone.0196999.s003.tif]

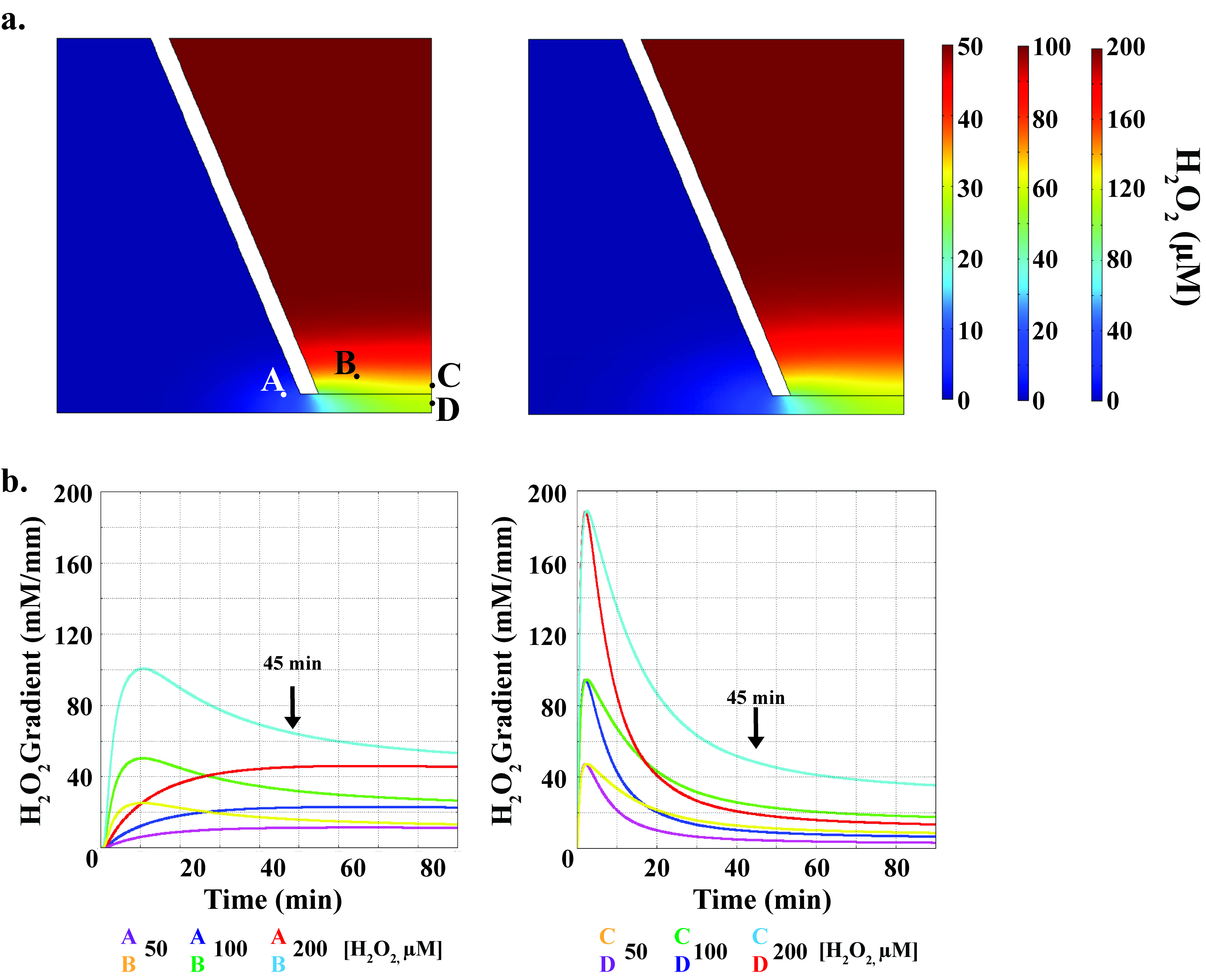

Supplement: S4 Fig — a. Comsol model heat maps. Four points (A-D) were recorded to estimate various hydrogen peroxide concentration gradients over time. Heat gradient maps were constructed for 45 minute (left) and 90 minute (right) gradients. Heat maps were generated using 50, 100, and 200 μM hydrogen peroxide starting concentrations. b. Hydrogen peroxide gradient curves over time. For each point (A-B, left; C-D, right), the hydrogen peroxide gradients were calculated over time to determine dynamics, steady-state conditions, and optimal measurement times (45 minutes). (TIF) [file pone.0196999.s004.tif]

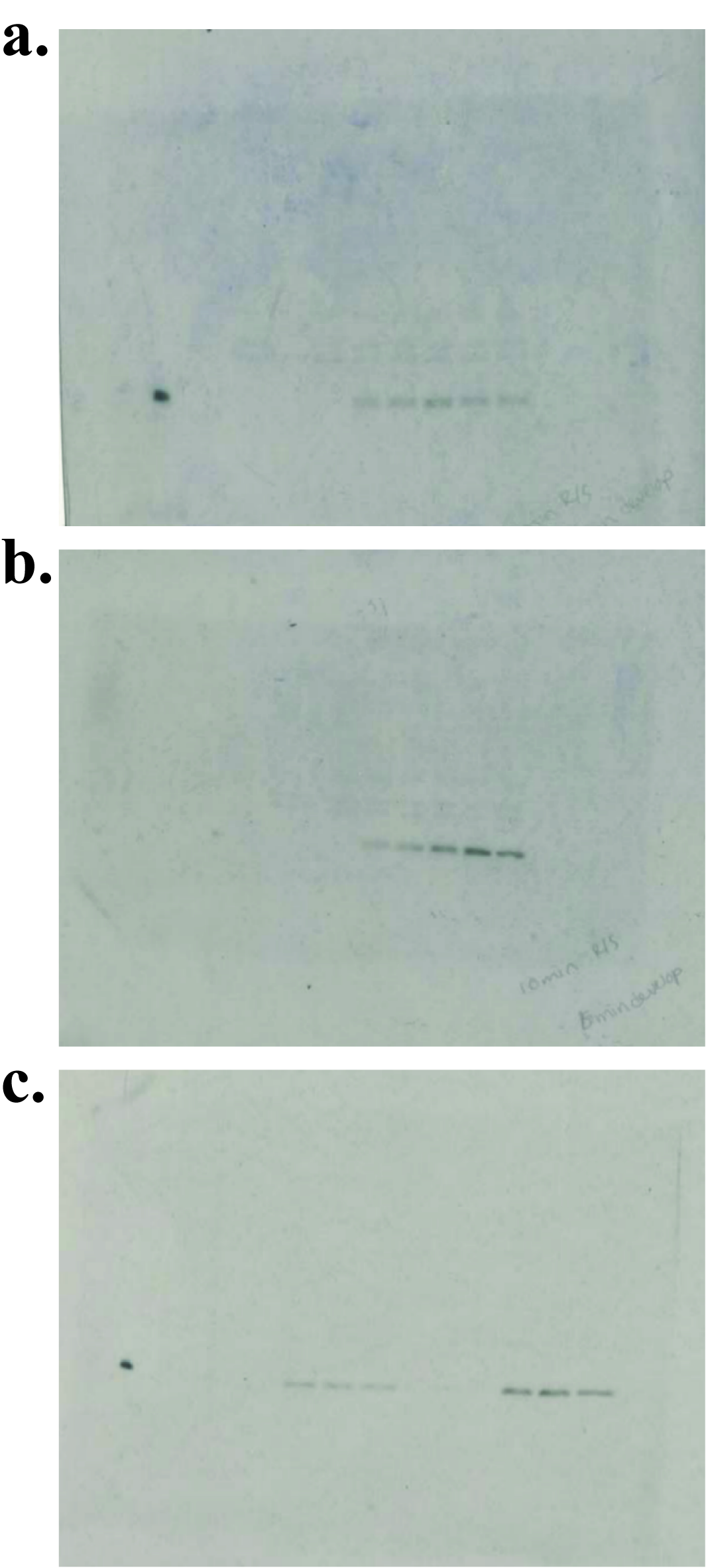

Supplement: S5 Fig — a. Hydrogen peroxide induction for 5 minutes. b. Hydrogen peroxide induction for 10 minutes. c. Hydrogen peroxide induction for 15 and 60 minutes. His6-CheZ and WT-pFZY1 were controls. (TIF) [file pone.0196999.s005.tif]

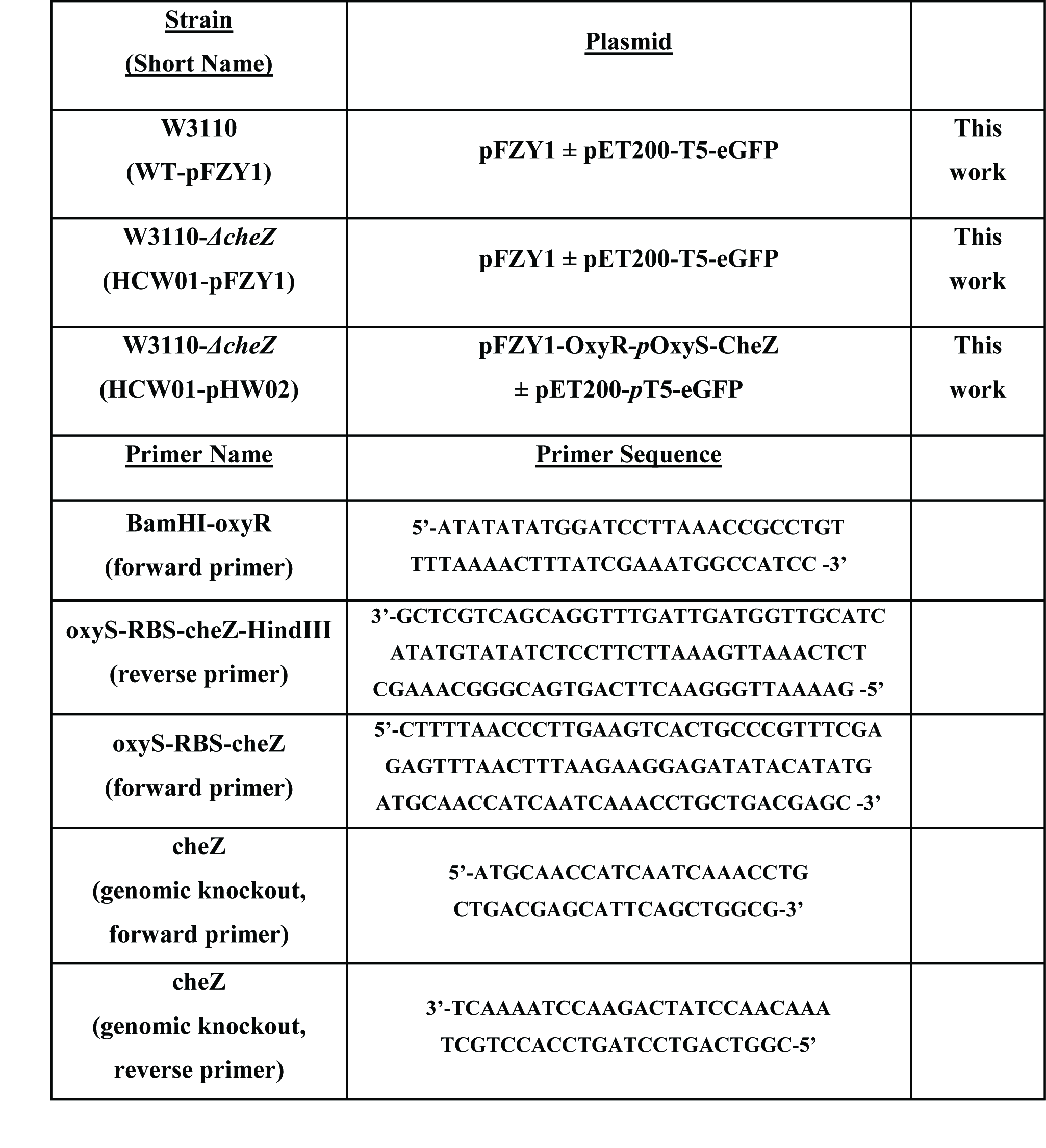

Supplement: S1 Table — (TIF) [file pone.0196999.s006.tif]
